# Cell Cycle Entry Control in Naïve and Memory CD8^+^ T Cells

**DOI:** 10.3389/fcell.2021.727441

**Published:** 2021-10-06

**Authors:** David A. Lewis, Tony Ly

**Affiliations:** ^1^Ashworth Laboratories, Institute of Immunology and Infectious Research, University of Edinburgh, Edinburgh, United Kingdom; ^2^Wellcome Centre for Cell Biology, University of Edinburgh, Edinburgh, United Kingdom; ^3^Centre for Gene Regulation and Expression, University of Dundee, Dundee, United Kingdom

**Keywords:** quiescence, T cell, proliferation, cell cycle, T cell activation

## Abstract

CD8^+^ T cells play important roles in immunity and immuno-oncology. Upon antigen recognition and co-stimulation, naïve CD8^+^ T cells escape from dormancy to engage in a complex programme of cellular growth, cell cycle entry and differentiation, resulting in rapid proliferation cycles that has the net effect of producing clonally expanded, antigen-specific cytotoxic T lymphocytes (CTLs). A fraction of activated T cells will re-enter dormancy by differentiating into memory T cells, which have essential roles in adaptive immunity. In this review, we discuss the current understanding of cell cycle entry control in CD8^+^ T cells and crosstalk between these mechanisms and pathways regulating immunological phenotypes.

## Introduction

**FIGURE 1:**
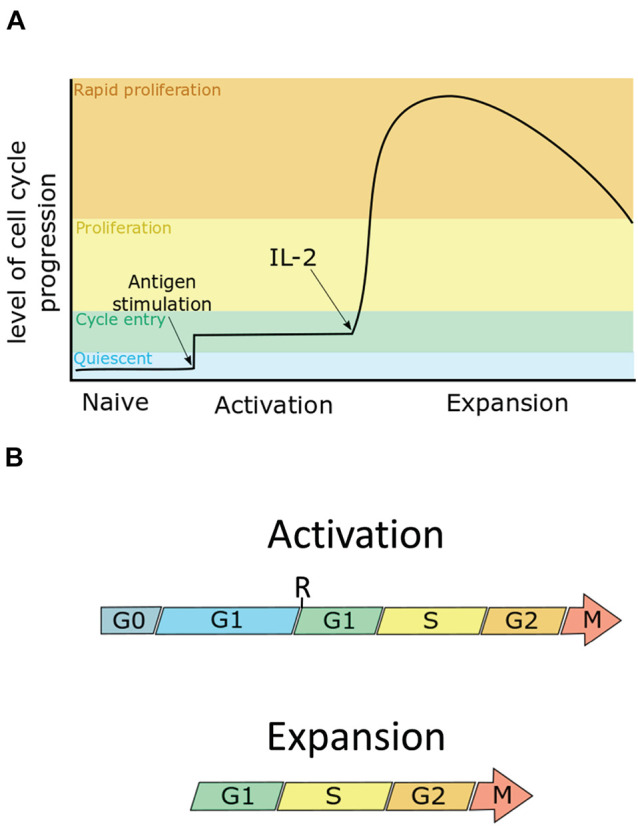
The cell cycle can be split into four phases: the first gap phase (G1), DNA synthesis phase (S), the second gap phase (G2), and mitosis (M). G1 can be further subdivided into pre- and post- restriction point G1 (pre-R G1, post-R G1). Quiescent cells are in a G0 state. **(A)** Naïve T cells are cells in G0. Upon maximum stimulation by T-cell receptor-antigen and co-stimulation, murine CD8^+^ T cells enter the cell cycle, going through only a few divisions until they receive the IL-2 signal, at which point they proliferate rapidly as they enter the expansion phase. **(B)** During activation, T cells enter an extended G1 phase, in which cells increase in size by ∼3–4-fold, entering their first S-phase within 30 h. During the expansion phase, cells then undergo rapid proliferation, dividing as quickly as once every 4 h, to clonally expand the antigen-specific T cell pool. Arrows indicate continuation beyond mitosis into the next G1.

CD8^+^ T cells are an important component of the acquired immune response. They have a role in limiting viral infection and cancer progression through recognising key viral/tumour antigens on the infected cell surface and targeting them for destruction (Kim and Ahmed, 2010). T cells are the product of central selection, a process in which newly developed progenitor T cells go through differentiation and numerous divisions, becoming lineage restricted toward the naïve CD8^+^ T cell phenotype. These naive T cells then lie dormant predominately within the lymph nodes (Carpenter and Bosselut, 2010). Naïve CD8^+^ T cells are activated by antigen presenting cells. The activation phase is characterised by major rewiring of their cellular proteomes and metabolism. Activated T cells then rapidly proliferate in what is known as the expansion phase (Figure 1A). During this phase, the doubling rate averages at 6–8 h with some studies demonstrating a peak doubling time of 4.5 h, or even 2 h (Kurts et al., 1997; Yoon et al., 2010). Also during this phase, T cells will begin to differentiate toward one of two T effector (T_*eff*_) phenotypes, the short lived effector cell (SLEC) which die during the subsequent contraction phase, and the memory progenitor cells (MPEC) which survive beyond the contraction phase. MPECs then differentiate toward one of the memory T cell phenotypes, e.g., the central memory T cell (T_*cm*_), and effector memory T cell (T_*em*_). The role of SLECs is to clear the infection, which is done by targeted release of cytolytic granzymes and perforins to infected cells. Following the clearance of infection, memory T cells remain in a state of dormancy, with T_*cm*_ localising to the lymph nodes, and T_*em*_ remaining in the periphery. Like naïve T cells, they await antigen stimulation in order to activate and proliferate, but have a higher antigenic threshold and more quickly initiate proliferation (Mehlhop-Williams and Bevan, 2014). This enables memory T cells to act as early responders to repeat infections.

What factors determine whether daughter cells from an activated naïve T cell move toward the MPEC or SLEC phenotype are a topic of active debate. It is generally agreed, however, that SLECs represent a fully differentiated form of T cell, which develop full effector function in exchange of proliferative potential and survival that typify MPECS. Complementing this model are studies that demonstrate differences in cell cycle progression between the phenotypes, with SLECs progressing at a much faster rate than MPEC (Kinjyo et al., 2015; Kretschmer et al., 2020) demonstrating a link between control of proliferation and differentiation.

In this review, we will first present an overview on cell cycle regulation based on various mammalian model systems. We then examine the pathways controlling CD8^+^ T cell differentiation and proliferation with reference to the cell cycle regulation pathways outlined prior and explore potential crosstalk between these two key processes.

## Cell Cycle Regulation

The cell cycle is divided into four phases (Figure 1B). In the first gap phase (G1), the cell prepares for DNA replication (S). This is followed by a second gap phase (G2) when the cell prepares for mitosis (M). Quiescent, or G0 cells enter the cell cycle by first transiting through G1. During G1, cells pass through a commitment step called the restriction point (R). Before R, cells are susceptible to cell cycle arrest by mitogen starvation. However, beyond R, progression through the cell cycle is mitogen-independent in human fibroblasts. The time cells spend in pre-R G1 is heterogeneous compared with the time spent in the remaining phases of the cell cycle (Zetterberg and Larsson, 1985; Zetterberg et al., 1995). Additionally mitogen starved cells which exit to G0 and are reintroduced to mitogen have been observed to take up to an additional 8 h to reach and complete mitosis compared to cells cultured in mitogen-replete conditions (Zetterberg and Larsson, 1991). Thus, G0, pre-R and post-R G1 can be considered distinct phases that are distinguished in their mitogen responsiveness and in the length of time needed to progress to S-phase. It should be noted, however, that it is as yet unclear if the restriction point as described in fibroblasts exists within T cells. This is due to the differences in mitogenic signalling within T cells which make the model established in fibroblasts more challenging to test in T cells.

Key proteins that control cell cycle progression include (1) the cyclin proteins, which rise and fall in abundance at key stages of cell cycle, (2) the cyclin-dependent kinases (CDKs) which become active when bound to their cognate cyclins, and (3) the CDK inhibitor proteins (CDKi) which control cell cycle progression at key check points in response to mitogen starvation or cellular stress (e.g., DNA damage).

### CDK-Rb-E2F

Cyclins, CDKs, and CDKis have key roles in regulating the activity of E2 factor F (E2F), a transcription factor that promotes the expression of cell cycle genes, including the cyclins and CDKs themselves.

In G0 and pre-R G1, E2F mediated transcription will be inhibited by pocket proteins Retinoblastoma protein (Rb), p107, and p130, preventing the cell from progressing to S phase (Stengel et al., 2009). An active kinase complex is formed by interaction of cyclin D with either CDK4 or CDK6, and this kinase activity mediates hyperphosphorylation of Rb, causing Rb to decouple from E2F, and thereby enable E2F to promote upregulation of its targets. Such targets include a large number of S phase-promoting proteins, including cyclin E and cyclin A. Cyclin E will form a complex with CDK2 which amongst other processes will contribute to Rb hyperphosphorylation, resulting in a positive feedback loop that activates E2F (Dyson, 1998). Recent work suggests that CDK4/6 regulates cellular processes other than those downstream of E2F. Cyclin D3-CDK6 phosphorylates key enzymes in the glycolysis pathway, diverting metabolites from glycolysis into the pentose phosphate pathway to promote redox balance (via production of NADPH) and cellular anabolism (e.g., by generating nucleotide precursors) (Wang et al., 2017).

Cyclin-dependent kinases activities can be inhibited by stoichiometric binding to CDKis, of which there are two main families: the INK4 family consisting of p16, p15, p18, and p19 which inhibit Cyclin D-CDK4/6 complex; and the Cip/Kip family which consists of p27, p21, and p57 that inhibit cyclin-CDK complexes, and are able to induce cell cycle arrest at G1 phase (Sherr and Roberts, 1999). The regulation of CDK4/6 complexes by the Cip/Kip family members are more complex. Cip/Kip proteins can promote the formation of cyclin-CDK complexes and in the absence of p21 or p27, cyclin D-CDK complexes do not form (Labaer et al., 1997; Cheng et al., 1999). Trimeric species containing p27 phosphorylated on tyrosine 74, cyclin D, and CDK4 retain kinase activity (Guiley et al., 2019). On the other hand, high levels of p21 inhibit CDK4 (Labaer et al., 1997). Cip/Kip proteins are inactivated by post-translational modification and degradation. For example, tyrosine phosphorylation on p27 by mitogen-activated tyrosine kinases disrupts the inhibitory interaction of p27 with CDK2 and promotes p27 degradation (Grimmler et al., 2007),

### Anaphase Promoting Complex/Cyclosome

Stability of cyclin, CDK and CDKi proteins are controlled post-translationally in a cell cycle regulated manner by E3 ubiquitin ligases. The anaphase promoting complex/cyclosome (APC/C) is a large, multi-subunit E3 ligase that targets many proteins, including cyclins and other E2F targets, for destruction in mitosis and G1. The APC/C has two co-activators, Cell Division Cycle (Cdc) 20 and Fizzy-related protein homolog-1 (Fzr1) (also known as Cdh1). The substrate adaptor functions of Cdc20 are primarily in mitosis, whereas Cdh1 is important in mitotic exit and G1. Indeed, inactivation of the APC/C-Cdh1 has been shown to be a second crucial step in promoting the transition from G1 to S (Cappell et al., 2016). Thus, proteins required for G1/S are upregulated by increased synthesis via transcription by E2F and increased stability via APC/C inactivation. APC/C inactivation in G1 is mediated by multiple mechanisms, including phosphorylation of Cdh1 (Kramer et al., 2000) by CDK2 (Lukas et al., 1999) and by the accumulation the pseudosubstrate inhibitor protein, F-box only protein 5 (Fbxo5) [also known as the early mitotic inhibitor 1 (Emi1)] (Cappell et al., 2018). Additionally, there are deubiquitinases (DUBs), including ubiquitin specific peptidase 37 (Usp37), which stabilise APC/C substrates by removing ubiquitin chains that would otherwise target these proteins for proteasomal destruction (Huang et al., 2011).

## Activation Phase (TCR Induced Proliferation)

T cell activation and proliferation requires three stimulation signals, antigenic stimulation via the T cell receptor (TCR), co-receptor signalling from professional antigen presenting cells via CD28, and cytokine stimulation primarily driven by interleukin (IL)-2. Stimulation of the TCR alone results in anergy. Anergic cells do not produce IL-2, fail to proliferate, and become unresponsive to further stimulation attempts. A dual signal of TCR and CD28 induces production of IL-2 and proliferation within the first 24 h. IL-2 stimulation through the IL-2 receptor (IL-2R) enables rapid proliferation as the cells enter the expansion phase (Mondino et al., 2006).

**FIGURE 2:**
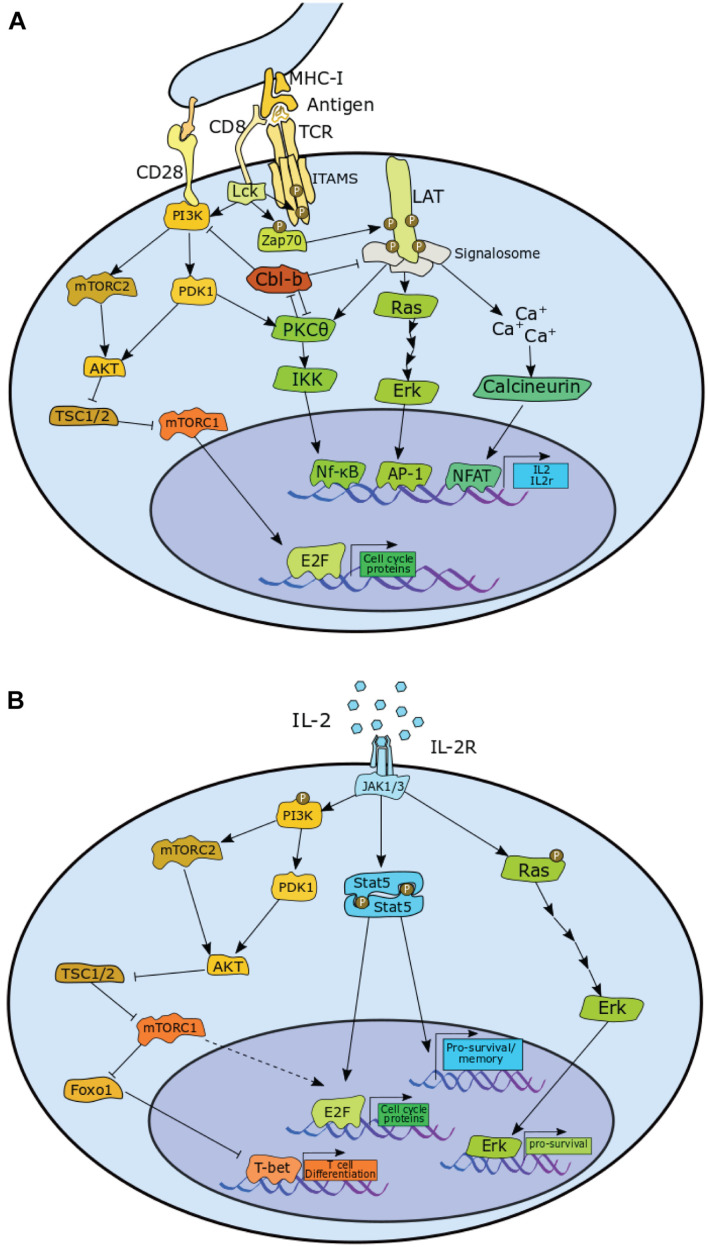
Pathways in CD8^+^ T cells that contribute to the proliferation via upregulation of E2F and pro-survival signals, or contribute toward differentiation in both **(A)** antigen induced activation phase and **(B)** the IL-2 mediated expansion phase CD8^+^ T cells. P, phosphorylation. A detailed description is provided in the main text.

As depicted in Figure 2A, TCR stimulation leads to phosphorylation of lymphocyte-specific protein tyrosine kinase (Lck), which in return phosphorylates the ζ chains of the TCR along the immunoreceptor tyrosine-based activation motifs (ITAMS). This enables recruitment of TCR-associated protein 70 (Zap70), which is subsequently phosphorylated by Lck. Zap70 then phosphorylates four key sites on the linker for activation of T cells (LAT) which allows for recruitment of proteins into the LAT signalosome. The downstream effect is activation of the Rat sarcoma (Ras)/extracellular signal-related kinase (Erk)/Activator protein 1 (AP-1) pathway, Protein kinase C–θ (PKCθ)/κB kinase (IKK)/nuclear factor-κB (Nf-κB) pathway, and the calcium-dependent Calcineurin/nuclear factor of activated T cells (NFAT) pathway (Brownlie and Zamoyska, 2013; Hwang et al., 2020). The transcription factors downstream of these pathways, NFAT, Nf-κB, and AP-1 all contribute to the transcription of *IL2* and also contribute to the transcription of *IL2RA*, which encodes the α-chain of the IL-2 receptor (CD25) (Liao et al., 2013).

Protein tyrosine kinase positively regulates phosphoinositide-dependent kinase 1 (PDK1) activity, which stimulates two major pathways: PKCθ/IKK/Nf-κB pathway and the Ak strain transforming (AKT)/ mammalian target of rapamycin complex 1 (mTORC1) pathway. Lck mediates this via activation of phosphoinositide 3-Kinase (PI3K), which is enhanced by co-receptor CD28 stimulation. PI3K is also responsible for enabling the assembly of mTOR complex 2 (mTORC2), which activates AKT, enabling it to target its substrates, such as the mTORC1 inhibitor, Hamartin (TSC1)/Tuberin (TSC2), thereby upregulating mTORC1 activity (Brownlie and Zamoyska, 2013; Jutz et al., 2016; Spolski et al., 2018; Hwang et al., 2020).

With TCR stimulation alone, however, the components of the LAT signalosome, as well as PKCθ are targeted for degradation by the E3-Ubiquitin ligase casitas B-lineage lymphoma proto-oncogene (Cbl)-b. Cbl-b also has the effect of reducing PI3K activity by preventing formation of phosphatidylinositol-3-phosphate (PIP3). The net effect of this is a sharp reduction in NFAT, NFκB, AP-1, and mTOR activity, resulting in the cell becoming anergic (Liu et al., 2014).

CD28 stimulation counteracts this effect by enhancing PKCθ activity, which promotes degradation of Cbl-b. Indeed, loss of PKCθ activity further decreases transcription factor activity downstream of LAT (Gruber et al., 2009), while knockout of CD28 leads to a reduction in proliferation (Li et al., 2004). Conversely, both Cbl-b deletion and Cbl-b inactive mutation enables T cell proliferation and production of IL-2 in the absence of CD28 signal. Loss of Cbl-b activity greatly enhances CD28-mediated proliferation and IL-2 production (Paolino et al., 2011). Consistent with a role in negatively regulating activation, Cbl-b also emerged as a top hit in a CRIPSR genetic screen for regulators of secondary activation in human CD8^+^ T cells (Shifrut et al., 2018).

Interestingly, there is no requirement for Lck in the activation of memory T cells upon secondary infection by LCMV *in vivo*. The frequency of antigen-specific T cells after secondary challenge to antigen was unaffected in memory cells depleted of Lck compared to Lck-replete cells, suggesting that proliferation is unaffected by Lck depletion. Re-challenged Lck-depleted cells also produced comparable levels of interferon (IFN)-γ to that of Lck-replete cells. This is in direct contrast to naïve cells depleted of Lck, which do not proliferate in response to antigen (Tewari et al., 2006). In addition, *ex vivo* memory T cells seem more reliant on cytokine signalling to induce proliferation, with antigen and CD28 stimulation alone proving insufficient to trigger proliferation in memory T cells (Cho et al., 1999).

### Mammalian Target of Rapamycin Complex, Cell Growth, and E2 Factor F

T cell receptor/CD28 stimulation of naïve cells is sufficient to initiate proliferation. TCR/CD28 stimulation leads to phosphorylation of Rb, expression of cyclin E, cyclin A, and CDK2, and the degradation of p27 (Appleman et al., 2000). Rapamycin, an inhibitor of mTOR, induces a severe delay in proliferation (D’Souza and Lefrançois, 2003), resulting in decreased levels of cyclin D3 and cyclin E. And while cyclin D3 levels recover 3–5 days following rapamycin treatment, cyclin E remains low, suggesting a dependence on mTOR for antigen-induced E2F activation. Although the exact mechanism is unknown, expression of a rapamycin resistant mutant of p70^*S6k*^ (also known as S6K) rescues E2F activity in rapamycin-treated cells; thereby demonstrating that mTOR activity promotes cell cycle progression by activating E2F through S6K (Brennan et al., 1999). In contrast to wild-type cells, proliferation of IL-2^–/–^ cells is arrested completely by rapamycin. In addition, stimulator of interferon genes (STING) activity reduces levels of cyclins A and E, and Cdk1, leading to a reduction in activation induced proliferation. STING inhibits mTOR, reduces S6K activity, reduces phosphorylation of eukaryotic translation initiation factor 4E (eIF4E)-binding protein (4E-BP1), and reduces STAT5 phosphorylation, leading to decreased levels of S-phase promoting proteins (Imanishi et al., 2019). Together these results suggest that IL-2 signalling contributes to proliferation in an mTOR-independent manner as outlined in Figure 2B. Interestingly, mTOR inhibition does not delay cells in the expansion phase (Colombetti et al., 2006).

Canonically there are three E2F isoforms which promote proliferation, E2F1-3, and that activation of these transcription factors are essential to cell cycle progression (Wu et al., 2001). However, it has been shown that in CD8^+^ T cells, E2F1-3 can have both positive and negative effects on cell cycle progression. Single knockout of either E2F1 or E2F2 results in reduction in proliferation, while a double knockout yields a hyperproliferative phenotype and reduced antigenic threshold (Murga et al., 2001; Zhu et al., 2001). In addition to this, CD8^+^ T cells deficient in E2F1 show much reduced activation induced cell death (Gao et al., 2004). CD8^+^ T cells are not the only cell line in which E2F behaves in a non-canonical fashion. In retinal cells Myc mediated proliferation has been shown to persist even in the absence of E2F1–3 proving these E2Fs to be largely non-essential for these cells (Chen et al., 2009), while in progenitor cell lines approaching terminal differentiation, E2F1-3 seem to have a cell cycle repressive role upon forming a complex with Rb (Chong et al., 2009). It is, however, difficult to say whether similar processes are occurring within CD8^+^ T cells post activation as the precise molecular regulators of E2F transcription targets have yet to be fully explored.

mTOR also upregulates the c-Myc transcription factor, which has a crucial role in T cell metabolism (Vartanian et al., 2011; Wang et al., 2011; Waickman and Powell, 2012) and expression of several cell cycle regulators, including p27, cyclins, and CDKs (Dang et al., 2006). T cells lacking c-Myc have defects in glycolysis, glutaminolysis, cell growth, and proliferation. Indeed, compared to wild type, c-Myc knockout cells show decreased levels of CDK4, CDK2, and Cdc25A (Wang et al., 2011). Furthermore, Raptor deficient cells that have impaired mTORC1 activity show reduced levels of key transcription factors including Myc, but also GA-binding protein alpha chain (Gabpa), yin yang (YY)1, and Sterol Regulatory Element Binding Transcription Factor (Srebf)1, all of which are associated with mitochondrial function (Tan et al., 2017).

### Cyclin-Dependent Kinase Regulation of T Cell Activation

CDKi proteins interfere with the pathways downstream of TCR stimulation. p27 is a key negative regulator of IL-2 production in anergic CD4^+^ T cells (Boussiotis et al., 2000). Ectopic expression of p27, but not p21, suppresses IL-2 production in CD4^+^ T cells. Depletion of p27 in stimulated Jurkat T cells enhances transcription of an IL-2 luciferase reporter construct and enhanced cellular AP-1 activity. The proposed mechanism is that p27 directly binds JAB1/COPS5, a subunit of the COP9 signalosome and positive regulates AP-1, inducing translocation of AP-1 from the nucleus to the cytoplasm (Tomoda et al., 1999), thereby suppressing IL-2 transcription (Boussiotis et al., 2000). Whether this effect extends to CD8^+^ T cells is yet to be determined.

There is significant interest in the roles of CDK4/6 in immunomodulation, and how small molecule inhibitors of CDK4/6 affect immune cell phenotypes. Recent data using CDK4/6 inhibitors (CDK4/6i) suggest a direct role of Cyclin-CDKs in controlling T cell differentiation. Three CDK4/6i are FDA-approved for HR+ breast cancer: palbociclib (PD-0332991), abemaciclib (LY2835219), and ribociclib (LEE011). Numerous clinical trials are ongoing for the treatment of other solid tumours (Álvarez-Fernández and Malumbres, 2020). Extensive reviews on this topic already exist and lie outside the scope of this review (Chaikovsky and Sage, 2018; Ameratunga et al., 2019). Interestingly, recent evidence suggests these inhibitors function similarly to the INK4 family of proteins by binding to monomeric CDK4/6 (Guiley et al., 2019). Proliferation of CD4^+^ Treg cells is inhibited by CDK4/6 inhibition whereas proliferation of CD8^+^ cells is relatively unaffected (Goel et al., 2017). Indeed, CD8^+^ T cell function can be augmented by CDK4/6 inhibition. Treatment of CD8^+^ T cells with CDK4/6 inhibitors enhances NFAT activity, increasing expression of CD25 and production of IL-2 and granzyme B, indicating that CDK6 may have a role in limiting T cell effector differentiation (Deng et al., 2018). Indeed, loss of CDK6 in T cells led to an increase in IL-2 production, and was also seen to impair type I interferon signalling events and increased metabolic processes resulting in enhanced ATP production and maximal respiration in addition to affecting proliferation (Klein et al., 2021).

In summary, T cell activation produces a signalling cascade that triggers a change in gene expression that promotes cell growth, increased anabolic metabolism, and stimulation of nascent CDK/E2F activities. The significant rewiring of the proteome and metabolism is important for supporting the next phase of CD8^+^ T cell differentiation, in which activated cells rapidly proliferate to clonally expand antigen-specific CTLs.

## Expansion Phase (Il-2 Driven Proliferation)

A key regulator of CD8^+^ T cell proliferation in the expansion phase is IL-2, a cytokine and potent T cell mitogen (Smith and Ruscetti, 1981) that is produced primarily by activated CD4^+^ and CD8^+^ T cells (Nelson, 2004). Upon activation, CD8^+^ T cells secrete IL-2 and express a high affinity IL-2 receptor subunit, CD25. Blockade of IL-2 leads to reduced proliferation (Mishima et al., 2017). While TCR stimulation alone has been shown to be sufficient to induce proliferation of CD8^+^ T cells, the absence of IL-2 signal results in suboptimal expansion and cell cycle slower progression (D’Souza and Lefrançois, 2003). In addition, the presence of IL-2 in culture reduces the minimum threshold of TCR signalling required to enter cell cycle (Au-Yeung et al., 2017).

Continuous exposure to IL-2 is essential for prolonged expansion. IL-2 starvation leads to reduced cell viability, loss of CD25 expression, and decreased cellular protein content, including many major cell cycle regulators: cyclin D2, cyclin D3, cyclin A, cyclin B1, cyclin B2, CDK4, CDK6, CDK1, p15, and p21. Notably, p27 is one of the few proteins to increase as a result of IL-2 starvation (Rollings et al., 2018). p27 is implicated in controlling cell cycle exit in the contraction phase. *In vivo* experiments utilising p27 KO CD8^+^ T cells continued to expand up to day 11 post immunisation, while WT began contraction by day 8. The effect of p27 is more pronounced for MPECs than SLECs. MPEC cells maintained their higher populations as late as 30 days post immunisation, while SLEC p27 knockouts generally returned to similar cell numbers as WT cells by day 15. This difference in response is likely due to MPECs expressing higher pro-survival factors and having a greater capacity for IL-2 production than SLECs (Singh et al., 2010).

Whereas studies consistently show that p27^–/–^ mice exhibit splenic and thymic hyperplasia (Fero et al., 1996; Kiyokawa et al., 1996; Nakayama et al., 1996), the data for p21^–/–^ mice are conflicting (Balomenos et al., 2000). Activated p21^–/–^, p27^–/–^ double knockout (DKO) splenocytes are hyperproliferative *in vitro*. Freshly isolated splenocytes from p21^–/–^ but not p27^–/–^ mice, had a higher frequency of CD25 expressing naïve CD4^+^ and CD8^+^ T cells. The percentage of CD25^+^ cells in activated DKO or p27^–/–^ splenocyte cultures were elevated by ∼10% relative to wild type, or to p21^–/–^ (Wolfraim et al., 2004), which may enhance T cell sensitivity to IL-2, and therefore mitogen-induced proliferation.

Downstream of IL-2 receptor activation is the STAT5 pathway (Moriggl et al., 1999a), which is known to positively regulate proliferation. STAT5 activation induces homeostatic proliferation in naïve CD8^+^ T cells, even in the absence of cytokine stimulation (Burchill et al., 2003), and in activated T cells, like mTOR, STAT5 enhances E2F activity by activating S6K (Lockyer et al., 2007). STAT5 KO T cells have reduced cell cycle regulators, including cyclin D2, cyclin D3, cyclin E, cyclin A, and CDK6 (Moriggl et al., 1999b) while inhibition of STAT5 results in a dose dependent loss of Cyclin E (Lai et al., 2009). All of these proteins play important roles in G1-S phase progression. STAT5 signalling upregulates levels of pro-survival proteins Bcl-2 and Bcl-X_*L*_. Additionally, STAT5 upregulates c-Myc, further implicating it as an important mediator of CD8^+^ T cell proliferation (Lord et al., 2000). Constitutively active STAT5 promotes homeostatic proliferation and survival of CD8^+^ T cells. Interestingly, STAT5 also enhances presence of T memory cell phenotype, increasing the frequency of cells expressing key memory markers including CD127, CD122, CD62L, and Bcl-2 (Hand et al., 2010).

As previously noted, CDK4/6 inhibitors are shown to enhance CD25 expression during activation. However, post expansion phase, cells that have been previously exposed to the inhibitors produce a greater pool of memory T cells by upregulating Max Dimerization Protein (Mxd)4, a negative regulator of Myc/Max formation (Heckler et al., 2021). Interestingly, this event seems to be independent of cell cycle arrest, suggesting a role for CDK4/6 in affecting T cell development (Heckler et al., 2021; Lelliott et al., 2021).

mTOR is also upregulated by IL-2 via the PI3K/AKT pathway. However, other pathways contribute more strongly to T cell proliferation in the expansion phase, as inhibiting mTOR in this phase does not arrest proliferation (Howden et al., 2019). Similarly, naïve CD8^+^ treated with AKT inhibitors responded less to stimulation, with proliferation severely reduced by day 2 of the culture in a dose dependent manner (Cho et al., 2013). If treated 3 days post activation, however, AKT inhibition resulted in little impact on proliferation (Macintyre et al., 2011).

Conversely, T cells expressing constitutively activated AKT were defective in memory T cell development *in vivo*. These cells have reduced CD127, CD122, CD62L, and Bcl-2 (Hand et al., 2010). CD127 reduction is predominately controlled by PI3K/AKT activity in a STAT5 independent way, as STAT5 knockout cells had little impact on CD127 (Xue et al., 2002). Constitutively active AKT cells also showed poor STAT5 phosphorylation in response to any cytokine stimulation, and as a result had a defect in homeostatic proliferation.

Taken together, these data suggest that a principal role of the IL2R/PI3K/AKT/mTOR pathway is the differentiation of T cells toward the SLEC phenotype, which occurs in parallel to IL2R/STAT5. The latter is the dominant pathway that promotes upregulation of cell cycle regulatory proteins and therefore proliferation of CD8^+^ T cells.

Cyclin E/A-CDK2 complexes phosphorylate numerous substrates, including targets outside of canonical cell cycle pathways that directly regulate DNA replication and mitosis. For example, cyclin E/A-CDK2 phosphorylates and inhibits Foxo1 within a number of cancer cell lines (Huang et al., 2006). In T cells, mTOR drives differentiation toward SLEC phenotype by phosphorylation of Foxo1, an inhibitor of SLEC phenotype transcription factor T-box expressed in T cells (T-bet) (Rao et al., 2010; Michelini et al., 2013; Pollizzi et al., 2015). Thus, CDK2-mediated inhibition of Foxo1 raises the intriguing possibility that CDK2 may also have a role in enhancing T-bet activity, and thus provide a mechanism by which high CDK2 expression can enhance differentiation. However, this has yet to be demonstrated in T cells.

## The Cell Cycle Control Protein Network in T Cells

**FIGURE 3:**
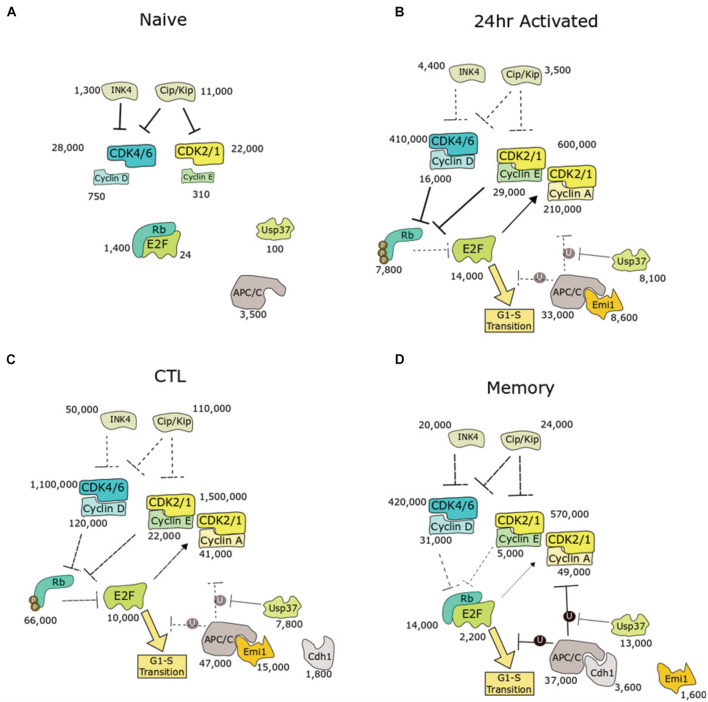
Biochemical pathway analysis of cell cycle entry in murine CD8^+^ T cells with protein copies measured by quantitative proteomics. Four populations were presented: **(A)** quiescent naive, **(B)** activation phase, **(C)** expansion phase toward SLEC phenotype, or **(D)** expansion phase toward MPEC phenotype. Numbers indicate copy number of each protein as determined by quantitative proteomics, acquired from Immpress (“ImmPRes.co.uk,” University of Dundee). For protein families (e.g., INK4, CIP/KIP, cyclins, and CDKs), summed copies are shown. E2F copies represent the sum of E2Fs 1–3. Median subunit copy number is shown for the APC/C. Mechanisms predicted to be dominant are highlighted in bold lines, whereas suppressed pathways are indicated by dotted lines. P, phosphorylation; U, ubiquitination.

T cell activation and their differentiation into effector and memory cells provides exemplar systems to study the G0 to G1 transition in non-immortalised cells in the physiological context of the adaptive immune response. The proteomes of these cells have been characterised to high depth using mass spectrometry-based proteomics, enabling detailed measurements of protein copies per cell in naïve and activated murine CD8^+^ T cells (Howden et al., 2019; Marchingo et al., 2020). The Immunological Proteomic Resource (ImmPRes) is an open access public resource consolidating proteomics experiments carried out on murine immune cell populations, including effector and memory CD8^+^ T cell states.^1^ We therefore used these publicly available data to evaluate the copies of key cell cycle proteins in naïve, activated (24 h), CTL (IL-2), and memory (IL-15) cell populations (Figure 3).

These data show that naïve cells have proportionally higher numbers of CDKis for each CDK (CIP/KIP and INK4) and very low abundance of cyclins. For example, naïve cells express 3,800 copies of p27 and less than 1,000 copies each of cyclin D2 and cyclin D3. In addition, levels of activating E2F transcription factors (20 copies) are two orders of magnitude lower than their stoichiometric inhibitor, Rb (1,400 copies). E2F activity is therefore strongly inhibited, which maintains naïve cells in quiescence. Interestingly, of the CIP/KIP family of proteins, p27 is higher expressed than p21 except during IL-2 mediated expansion, supporting a role of p27 over p21 as a regulator of quiescence and homeostatic proliferation in T cells.

Upon activation, E2F levels increase 500-fold to 14,000 copies, contributing to a gene expression programme that promotes G1/S progression. For example, levels of CDK1/2/4/6, cyclin D, cyclin E, and cyclin A are all upregulated. Intriguingly, while Rb levels increase in activated T cells to 7,800 copies. There is a 3-fold increase in cyclin D2, and 8-fold increase in cyclin D3, and around a 30-fold increase in CDK4/6 copies, while p27 decreases 19-fold. Interestingly, CD8^+^ T cells can bypass CDK4/6 inhibition (Goel et al., 2017), leading to slowed proliferation (Heckler et al., 2021; Lelliott et al., 2021), but not arrest. The major changes observed copies in the Rb-E2F pathway may contribute to this resistance to G1 arrest by CDK4/6 inhibitors.

Inactivation of the APC/C is another mechanism to promote G1/S transition (Cappell et al., 2018). Median copies of the core APC/C subunits increase from 3,500 copies in naïve to 33,000 copies in activated T cells. Interestingly, Cdh1, the co-activator and substrate adaptor subunit of APC/C during G1, is undetectable in naïve and activated T cells. Likewise, Emi1 is undetectable in naïve. However, Emi1, an E2F target, increases to 8,600 copies in activated T cells.

Interleukin-2-mediated differentiation into CTLs is accompanied by a nearly 10-fold increase in Rb levels relative to 24 h activated T cells. Unlike newly activated T cells, which have nearly 2-fold excess E2F to Rb, CTLs contain far higher copies of Rb (66,000) to E2F (10,000). Thus, in CTLs there are sufficient levels of Rb, when not phosphorylated by CDK, to suppress all copies of E2F. However, copies of cyclins and CDKs are much higher in CTLs, leading to high activity of CDKs, which phosphorylate and inactivate Rb. These two mechanisms of E2F activation, Rb phosphorylation and excess E2F, suggest that newly activated T cells and CTLs may have differential sensitivity to pharmacological inhibition of CDK2/4/6.

Memory T cells produced by culture with IL-15 proliferate slower. In contrast to CTLs, memory T cells have reduced levels of cyclins and CDK2/4/6. However, like CTLs, copies of Rb (14,000) exceed copies of E2F (2,200). This may make memory T cells more sensitive toward Rb-mediated inhibition of E2F activity compared to naïve and 24-activated T cells. Memory T cells have increased copies of APC-C/Cdh1 (3,600) compared to CTLs, which also may contribute to extending G1 phase by keeping cyclin levels low.

## Concluding Remarks

Immune cells play critical roles in normal physiology, pathology, and the development of new therapies to treat disease. Understanding fundamental mechanisms for how proliferation is controlled in T cells will be important in making advances in these areas of immunological research. This is highlighted by recent studies showing that CDK4/6 inhibitors affect proliferation of CD4^+^ T_reg_ cells, but not CTLs (Goel et al., 2017), and enhance functions of checkpoint-activated CD8^+^ T cells (Deng et al., 2018). These results suggest that there are major differences between CD4^+^ and CD8^+^ T cells in G1 control, which will be interesting to explore in future studies. Deeper insights into how the cell cycle control network is structured in different immune subsets may provide important clues into the mode of action of these inhibitors on immune cells and an opportunity to identify new targets for modulating immune cell proliferation and function.

## Author Contributions

DL prepared the figures. DL and TL wrote the manuscript. Both authors contributed to the article and approved the submitted version.

## Conflict of Interest

The authors declare that the research was conducted in the absence of any commercial or financial relationships that could be construed as a potential conflict of interest.

## Publisher’s Note

All claims expressed in this article are solely those of the authors and do not necessarily represent those of their affiliated organizations, or those of the publisher, the editors and the reviewers. Any product that may be evaluated in this article, or claim that may be made by its manufacturer, is not guaranteed or endorsed by the publisher.

## Footnotes

1
http://www.immpres.co.uk/
